# Recurrence of Atrial Fibrillation in Patients With New-Onset Postoperative Atrial Fibrillation After Coronary Artery Bypass Grafting

**DOI:** 10.1001/jamanetworkopen.2024.1537

**Published:** 2024-03-07

**Authors:** Florian E. M. Herrmann, Amar Taha, Susanne J. Nielsen, Andreas Martinsson, Emma C. Hansson, Gerd Juchem, Anders Jeppsson

**Affiliations:** 1 Department of Molecular and Clinical Medicine, Institute of Medicine, Sahlgrenska Academy, University of Gothenburg, Gothenburg, Sweden; 2 Department of Cardiac Surgery, LMU University Hospital, LMU Munich, Munich, Germany; 3 DZHK (German Centre for Cardiovascular Research) Partner Site Munich Heart Alliance, Munich, Germany; 4 Department of Cardiology, Region Västra Götaland, Sahlgrenska University Hospital, Gothenburg, Sweden; 5 Department of Cardiothoracic Surgery, Region Västra Götaland, Sahlgrenska University Hospital, Gothenburg, Sweden

## Abstract

**Importance:**

New-onset postoperative atrial fibrillation (POAF) occurs in approximately 30% of patients undergoing coronary artery bypass grafting (CABG). It is unknown whether early recurrence is associated with worse outcomes.

**Objective:**

To test the hypothesis that early AF recurrence in patients with POAF after CABG is associated with worse outcomes.

**Design, Setting, and Participants:**

This Swedish nationwide cohort study used prospectively collected data from the SWEDEHEART (Swedish Web System for Enhancement and Development of Evidence-Based Care in Heart Disease Evaluated According to Recommended Therapies) registry and 3 other mandatory national registries. The study included patients who underwent isolated first-time CABG between January 1, 2007, and December 31, 2020, and developed POAF. Data analysis was performed between March 6 and September 16, 2023.

**Exposure:**

Early AF recurrence defined as an episode of AF leading to hospital care within 3 months after discharge.

**Main Outcomes and Measures:**

The primary outcome was all-cause mortality. Secondary outcomes included ischemic stroke, any thromboembolism, heart failure hospitalization, and major bleeding within 2 years after discharge. The groups were compared with multivariable Cox regression models, with early AF recurrence as a time-dependent covariate. The hypothesis tested was formulated after data collection.

**Results:**

Of the 35 329 patients identified, 10 609 (30.0%) developed POAF after CABG and were included in this study. Their median age was 71 (IQR, 66-76) years. The median follow-up was 7.1 (IQR, 2.9-9.0) years, and most patients (81.6%) were men. Early AF recurrence occurred in 6.7% of patients. Event rates (95% CIs) per 100 patient-years with vs without early AF recurrence were 2.21 (1.49-3.24) vs 2.03 (1.83-2.25) for all-cause mortality, 3.94 (2.92-5.28) vs 2.79 (2.56-3.05) for heart failure hospitalization, and 3.97 (2.95-5.30) vs 2.74 (2.51-2.99) for major bleeding. No association between early AF recurrence and all-cause mortality was observed (adjusted hazard ratio [AHR], 1.17 [95% CI, 0.80-1.74]; *P* = .41). In exploratory analyses, there was an association with heart failure hospitalization (AHR, 1.80 [95% CI, 1.32-2.45]; *P* = .001) and major bleeding (AHR, 1.92 [1.42-2.61]; *P* < .001).

**Conclusions and Relevance:**

In this cohort study of early AF recurrence after POAF in patients who underwent CABG, no association was found between early AF recurrence and all-cause mortality. Exploratory analyses showed associations between AF recurrence and heart failure hospitalization, oral anticoagulation, and major bleeding.

## Key Points

**Question:**

Is early atrial fibrillation (AF) recurrence in patients with new-onset postoperative atrial fibrillation (POAF) after coronary artery bypass grafting (CABG) associated with worse outcomes?

**Findings:**

In this cohort study of 10 609 patients with POAF after CABG, early AF recurrence was not associated with an increased risk of all-cause mortality (primary endpoint), stroke, or thromboembolism within 2 years after discharge. Early recurrence was associated with an increased risk of heart failure hospitalization and major bleeding.

**Meaning:**

These findings suggest that patients with early AF recurrence after CABG with POAF constitute a risk group for which follow-up is advisable.

## Teaser

This cohort study examines the association between early atrial fibrillation (AF) recurrence and worse outcomes in patients with postoperative AF after coronary artery bypass grafting (CABG).

## Introduction

New-onset postoperative atrial fibrillation (POAF) is common after coronary artery bypass grafting (CABG), with a reported incidence of 21% to 34%.^1,2,3^ In most studies on this condition, POAF after CABG is associated with an increased short-term and long-term risk of stroke,^4^ thromboembolism, and heart failure hospitalization.^5^ Although POAF is thought to be a transient event, its negative association with long-term morbidity and mortality^6,7^ suggests a possible sentinel role, making POAF after cardiac surgery akin to a marker of generally enhanced cardiovascular risk.

Only a few previous studies have reported on atrial fibrillation (AF) recurrence in patients with POAF after CABG.^5,8,9^ These studies showed that patients with POAF after CABG have a substantial risk of AF recurrence during the first postoperative years. Taha et al^5^ reported a 4-fold increased adjusted risk of AF in patients with POAF after CABG during 8 years of follow-up compared with those without POAF, and Ahlsson et al^8^ reported an 8-fold increased risk. To our knowledge, no previous investigations of AF recurrence in the POAF population have evaluated the potential association between recurrence and outcomes.

In this large registry study of patients with POAF after CABG, we tested the hypothesis that early AF recurrence is associated with outcomes. We investigated the association of AF recurrence within the first 3 months after discharge with all-cause mortality, ischemic stroke, any thromboembolism, heart failure hospitalization, and major bleeding within the first 2 years after discharge.

## Methods

This cohort study was approved by the Swedish Ethical Review Authority, which waived the need for individual patient consent because registry data were used. The study was performed in accordance with the Declaration of Helsinki^10^ and followed the Strengthening the Reporting of Observational Studies in Epidemiology (STROBE) reporting guideline.

### Study Design and Patient Cohort

**Figure 1.**
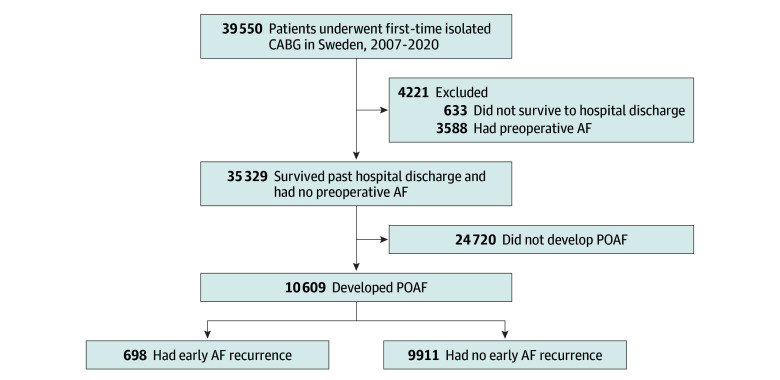
Flowchart of Population Selection AF indicates atrial fibrillation; CABG, coronary artery bypass grafting; POAF, new-onset postoperative atrial fibrillation.

This study drew its population from the Swedish Cardiac Surgery Registry,^11^ which is part of the SWEDEHEART (Swedish Web System for Enhancement and Development of Evidence-Based Care in Heart Disease Evaluated According to Recommended Therapies) registry.^12^ Eligible patients were identified and were included if they were aged 18 years or older and underwent first-time isolated CABG between January 1, 2007, and December 31, 2020. Patients who did not survive to discharge and those with AF prior to surgery were excluded (Figure 1). Patients were considered to have POAF if they (1) were reported in the SWEDEHEART registry or the National Patient Register^13^ to have developed new-onset AF during the index hospitalization or (2) underwent postoperative electrical cardioversion during the index hospitalization, as reported in the National Patient Register. Patients were considered to have experienced AF recurrence if they had experienced POAF during the index CABG hospitalization and presented to a hospital or hospital-affiliated outpatient clinic after the CABG index hospitalization, with AF recorded as the main diagnosis in the National Patient Register.

Early AF recurrence was defined as AF recurrence within the first 3 months after discharge from the CABG index hospitalization, similar to early AF recurrence after AF ablation.^14^ All included patients were followed until death, emigration, or the end of the study period (December 31, 2020). No patients were lost to follow-up. Patients who emigrated were censored at the time of emigration.

### Data Sources

Baseline characteristics, including preoperative AF, were collected from the SWEDEHEART registry^11^ and the National Patient Register.^13,15^ Postoperative outcome data were collected from the National Patient Register. Information about postoperatively dispensed medications was collected from the Swedish National Prescribed Drug Register. Mortality data were collected from the National Cause of Death Register. Information from each of the aforementioned registries was linked using the unique personal identification number allocated to each Swedish citizen at birth or after immigration.

The Swedish Cardiac Surgery Registry has documented all cardiac surgical procedures in Sweden since 1992. The National Patient Register has documented all diagnoses associated with hospital admissions since 1987 and hospital outpatient visits since 2001. This registry has full coverage and a validity of 85% to 95%, depending on the diagnosis.^15^ Codes from the *International Classification of Diseases*, *Tenth Revision* (*ICD-10*), were used to identify diagnoses and outcome events (eTable 1 in Supplement 1). The National Prescribed Drug Register has documented information regarding all dispensed prescription drugs since July 2005 using Anatomical Therapeutic Chemical classification codes (eTable 2 in Supplement 1). The Swedish Cause of Death Register has collected all data regarding deaths in Sweden since 1961.^16^

### Outcome Variables

The primary outcome variable in this study was all-cause mortality within 2 years of discharge after CABG. Secondary outcome variables were ischemic stroke, any thromboembolic event (including ischemic stroke, transient ischemic attack [TIA], or systemic embolism), heart failure hospitalization, and major bleeding. Outcome events were considered as having occurred if they led to a hospital admission or death.

### Statistical Analysis

The cumulative incidence of AF recurrence after CABG was calculated using regression modeling, taking into account the competing risk of death.^17^ Cumulative incidences are presented with 95% CIs. We compared patients with vs without early AF recurrence after surgery. Descriptive statistics are presented as medians and IQRs for continuous variables and as counts and percentages for categorical variables. For the comparison of baseline characteristics, the Wilcoxon test was used for continuous data and the Pearson χ^2^ test was used for categorical data. The median follow-up time was calculated using the reverse Kaplan-Meier method.^18^ Associations between early AF recurrence and outcome events were assessed by observing AF recurrence as a time-dependent covariate updated continuously during follow-up. Applying this methodology, outcome events were only interpreted as having occurred in a patient with early AF recurrence if a patient experienced the AF recurrence event prior to or on the day of the outcome events. Any outcome events occurring prior to AF recurrence events were interpreted as having occurred in a patient without early AF recurrence.

The association between early AF recurrence and outcomes was investigated using Cox proportional hazards models. Further outcome-associated variables were included in models used for the calculation of adjusted hazard ratios (AHRs; eTable 3 in Supplement 1 lists the variables included in each model). The proportional hazards assumption was evaluated by visual inspection of residual graphs and investigation of the Schoenfeld individual test. The assumption was not violated in any model. Event rates were calculated based on the time-to-event data as prepared for the Cox models respecting the time dependence of AF recurrence and subsequently only attributing time and events to early AF recurrence after AF recurrence events.

A sensitivity analysis was performed to assess the robustness of the results depending on an adapted interpretation of event-specific time at risk as related to the time-dependent covariate early AF recurrence (eMethods in Supplement 1). E values were calculated to evaluate the influence of confounding due to unknown or unmeasured variables.^19,20^ The calculations provide a value that describes what the magnitude of the association of the unmeasured confounder would have to be with each of the 2 investigated variables to explain away the observed association between the 2 variables. Hypothesis testing was performed with a significance level of *P* < .05. All statistical analyses were performed using R, version 4.2.2 (R Project for Statistical Computing),^21^ and the R packages *survival*, *survminer*, *tableone*, *tidycmprsk*, and *ggplot2*. Data analysis was performed between March 6 and September 16, 2023.

## Results

### Study Cohort

During the study period, 39 550 patients underwent first-time isolated CABG. A total of 633 (1.6%) did not survive to be discharged from the hospital and 3588 (9.1%) had a history of AF prior to surgery (Figure 1). Of the remaining 35 329 patients, 10 609 (30.0%) developed POAF after surgery and were therefore included in the study cohort. Forty-two patients (0.4%) emigrated during follow-up and were censored at the time of emigration. The median age of the POAF study cohort was 71 (IQR, 66-76) years; 81.6% of patients were men and 18.4% were women. A total of 6.5% of patients had an ischemic stroke, 6.3% had a TIA, and 56.2% had acute coronary syndrome before surgery. Also, 30.3% of the patients had diabetes and 13.6% had heart failure. Baseline patient characteristics are presented in Table 1. The median follow-up was 7.1 (IQR, 2.9-9.0) years.

**Table 1.** Baseline Clinical Characteristics of Patients With New-Onset POAF After CABG With and Without Early AF Recurrence^a^

Characteristic	Early AF recurrence (n = 698)^b^	No early AF recurrence (n = 9911)	*P* value
Sex			
Female	114 (16.3)	1837 (18.5)	.16
Male	584 (83.7)	8074 (81.5)	.16
Age, median (IQR), y	70 (66-76)	70 (66-76)	.42
Acute coronary syndrome	385 (55.2)	5578 (56.3)	.59
Previous percutaneous coronary intervention	110 (15.8)	1736 (17.5)	.26
Hypertension	519 (74.4)	7434 (75.0)	.73
Diabetes	164 (23.5)	3049 (30.8)	<.001
Heart failure	89 (12.8)	1359 (13.7)	.51
Left ventricular ejection fraction <50%	189 (27.2)	3137 (31.8)	.01
Previous ischemic stroke	37 (5.3)	654 (6.6)	.21
Previous hemorrhagic stroke	4 (0.6)	75 (0.8)	.75
Previous transient ischemic attack	43 (6.2)	625 (6.3)	.94
Previous systemic embolism	3 (0.4)	53 (0.5)	.92
Peripheral vascular disease	64 (9.2)	1079 (10.9)	.18
CHA_2_DS_2_-VASc score			
≥2	665 (95.3)	9449 (95.3)	>.99
≥4	350 (50.1)	5343 (53.9)	.06
Kidney failure	23 (3.3)	396 (4.0)	.41
Kidney replacement therapy	4 (0.6)	52 (0.5)	>.99
Chronic respiratory disease	60 (8.6)	1091 (11.0)	.06
Liver disease	3 (0.4)	94 (0.9)	.24
History of cancer	116 (16.6)	1711 (17.3)	.70

Abbreviations: AF, atrial fibrillation; CABG, coronary artery bypass grafting; CHA_2_DS_2_-VASc, congestive heart failure, hypertension, age, diabetes, prior stroke or transient ischemic attack or thromboembolism, vascular disease, age, and sex category; POAF, postoperative atrial fibrillation.

^a^ Unless indicated otherwise, values are presented as No. (%) of patients.

^b^ Recurrence within 3 months of discharge.

### Incidence of AF Recurrence

**Figure 2.**
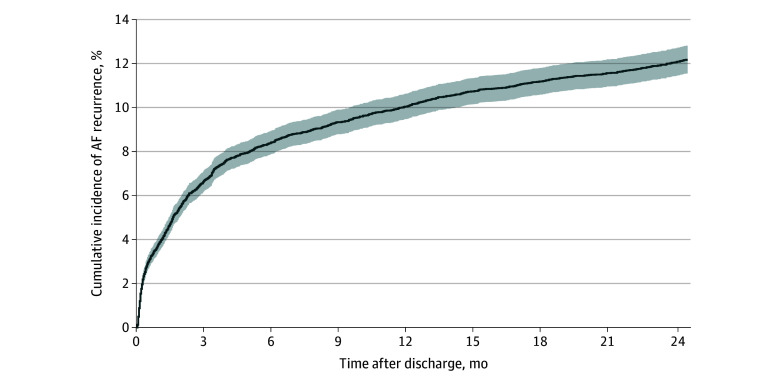
Atrial Fibrillation (AF) Recurrence Within 24 Months of Discharge in Patients With Postoperative Atrial Fibrillation (POAF) After Coronary Artery Bypass Grafting (CABG) Cumulative incidence of AF recurrence, adjusted for the competing risk of death, within 24 months of discharge in patients with new-onset POAF after CABG. Shaded area indicates 95% CIs.

**Figure 3.**
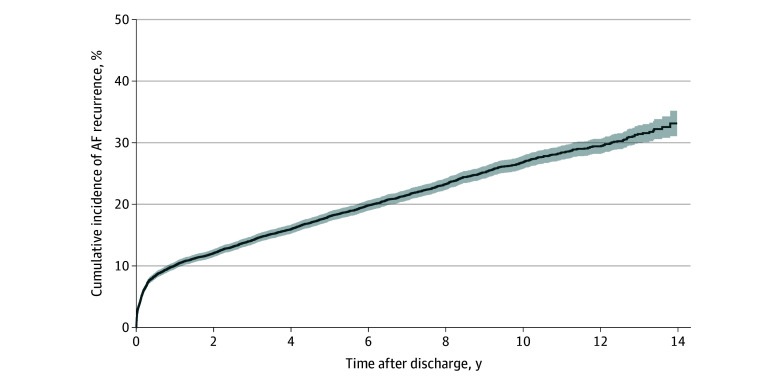
Atrial Fibrillation (AF) Recurrence Within 14 Years of Discharge in Patients With Postoperative Atrial Fibrillation (POAF) After Coronary Artery Bypass Grafting (CABG) Cumulative incidence of AF recurrence, adjusted for the competing risk of death, within 14 years of discharge in patients with new-onset POAF after CABG. Shaded area indicates 95% CIs.

The cumulative incidence (95% CI) of AF recurrence requiring hospital care was 3.8% (3.5%-4.2%) at 1 month, 6.7% (6.2%-7.1%) at 3 months, 10.0% (9.5%-10.6%) at 1 year, and 12.1% (11.4%-12.7%) at 2 years after discharge (Figure 2). Five years after discharge, 18.1% of patients (95% CI, 17.3%-18.8%) had experienced at least 1 AF recurrence event compared with 26.8% (25.8%-27.9%) at 10 years and 33.1% (31.1%-35.2%) at 14 years after discharge (Figure 3 and eTable 4 in Supplement 1). Cumulative incidences did not differ when comparing different time frames in which patients underwent CABG (eFigure in Supplement 1). The median time to AF recurrence was 15.8 (IQR, 1.9-58.2) months in the whole study cohort and 21 (IQR, 6-49) days in patients with early recurrence.

### Drug Dispensing Data

The proportion of patients with early AF recurrence who had been dispensed an oral anticoagulant increased from 33.3% directly after discharge to 83.2% at 1 month and 90.0% at 3 months; the corresponding values for patients without early AF recurrence were 20.7%, 29.0%, and 31.9%, respectively. The prevalence of antiplatelet therapy at 6 months after discharge was 81.5% in patients with early AF recurrence and 92.1% in patients without early AF recurrence.

The proportion of patients dispensed β-blockers during the first 6 postoperative months was 93.8% in patients with early AF recurrence and 93.6% in patients without early AF recurrence. The prevalence of amiodaron was 22.1% in patients with early AF recurrence and 14.0% in patients without early AF recurrence. The prevalence of digoxin was 13.5% in patients with early AF recurrence and 2.7% in patients without early AF recurrence. Finally, the prevalence of sotalol was 8.5% in patients with early AF recurrence and 7.9% in patients without early AF recurrence.

### Outcomes With and Without Early AF Recurrence

#### All-Cause Mortality

A total of 397 patients died during the first 2 years after discharge. The mortality rate (95% CI) was 2.21 (1.49-3.24) per 100 patient-years with early AF recurrence and 2.03 (1.83-2.25) without early AF recurrence. After multivariable adjustment, early AF recurrence was not associated with increased mortality risk (AHR, 1.17 [95% CI, 0.80-1.74]; *P* = .41) (Table 2).

**Table 2.** Event Rates and HRs Within 2 Years of Discharge in Patients With New-Onset POAF After Coronary Artery Bypass Grafting With and Without Early AF Recurrence

Outcome	No. of events	Person-years at risk	Event rate (95% CI)^a^	Early AF recurrence vs no early AF recurrence, HR (95% CI)^b^		*P* value
				Unadjusted	Adjusted^c^	
All-cause mortality						
AF recurrence	27	1222	2.21 (1.49-3.24)	1.12 (0.75-1.65)	1.17 (0.80-1.74)	.41
No AF recurrence	370	18 221	2.03 (1.83-2.25)			
Ischemic stroke						
AF recurrence	19	1192	1.59 (0.99-2.53)	0.91 (0.57-1.45)	0.98 (0.62-1.56)	.93
No AF recurrence	366	17 837	2.05 (1.85-2.27)			
Any thromboembolism						
AF recurrence	22	1185	1.86 (1.20-2.85)	0.77 (0.50-1.19)	0.86 (0.56-1.32)	.49
No AF recurrence	494	17 688	2.79 (2.56-3.05)			
Heart failure hospitalization						
AF recurrence	45	1143	3.94 (2.92-5.28)	1.72 (1.27-2.34)	1.80 (1.32-2.45)	.001
No AF recurrence	492	17 611	2.79 (2.56-3.05)			
Major bleeding						
AF recurrence	46	1159	3.97 (2.95-5.30)	1.80 (1.33-2.44)	1.92 (1.42-2.61)	<.001
No AF recurrence	484	17 660	2.74 (2.51-2.99)			

Abbreviations: AF, atrial fibrillation; HR, hazard ratio; POAF, postoperative atrial fibrillation.

^a^ Calculated for a maximum of 2 postoperative years and calculated as events per 100 patient-years. Both event rate and HR calculations observed the time dependence of the variable AF recurrence.

^b^ Recurrence within 3 months of discharge.

^c^ Adjusted for patient characteristics and comorbidities.

#### Stroke or Any Thromboembolism

A total of 385 ischemic strokes and 516 thromboembolic events (composite of ischemic stroke, TIA, and systemic embolism) occurred within the first 2 years after discharge. The ischemic stroke rate (95% CI) was 1.59 (0.99-2.53) events per 100 patient-years in patients with early AF recurrence and 2.05 (1.85-2.27) without early AF recurrence. After multivariable adjustment, early AF recurrence was not associated with ischemic stroke risk (AHR, 0.98 [95% CI, 0.62-1.56]; *P* = .93). The rate (95% CI) of any thromboembolism was 1.86 (1.2-2.85) events per 100 patient-years with early AF recurrence and 2.79 (2.56-3.05) without early AF recurrence. No association was observed between early AF recurrence and thromboembolic risk (AHR, 0.86 [95% CI, 0.56-1.32]; *P* = .49).

#### Heart Failure Hospitalization

A total of 537 heart failure hospitalizations occurred within the first 2 years after discharge. The rate (95% CI) for heart failure hospitalization was 3.94 (2.92-5.28) events per 100 patient-years with early AF recurrence and 2.79 (2.56-3.05) without early AF recurrence. After multivariable adjustment, early AF recurrence was associated with an increased risk of heart failure hospitalization (AHR, 1.80 [95% CI, 1.32-2.45]; *P* = .001).

#### Major Bleeding

A total of 530 bleeding events occurred within the first 2 years after discharge. The event rate (95% CI) for major bleeding was 3.97 (2.95-5.30) events per 100 patient-years with early AF recurrence and 2.74 (2.51-2.99) without early AF recurrence. After multivariable adjustment, early AF recurrence was associated with an increased risk of major bleeding (AHR, 1.92 [95% CI, 1.42-2.61]; *P* < .001).

### Sensitivity Analysis

Results of the sensitivity analyses are presented in eTable 5 in Supplement 1. The results supported the main analysis. E values for the association of heart failure hospitalization and major bleeding with AF recurrence were 3.0 and 3.2, respectively, for the estimates and 2.0 and 2.2, respectively, for the lower confidence limits.

## Discussion

In this nationwide observational study of Swedish patients with new-onset POAF after CABG, early AF recurrence leading to hospital care was common, occurring within 3 months in 6.7% of patients. Early AF recurrence was not associated with an increased risk of all-cause mortality (the primary endpoint). The exploratory analyses suggested that early AF recurrence was associated with heart failure hospitalization, increased dispensation of oral anticoagulants, and an increased risk of major bleeding events during the first 2 years after discharge.

The observation that “atrial fibrillation begets atrial fibrillation” describes that electrical remodeling caused by AF results in a condition favoring continued AF.^22^ Previous studies have suggested that POAF may favor later development of AF. Depending on the study, variable incidence of AF recurrence after CABG has been observed. Butt et al^23^ reported that 8.7% of all hospitalizations in the first year after CABG in Denmark are due to AF. Their study did not differentiate between patients who had experienced POAF and those who had not. In a community-based study investigating patients who had undergone CABG, valvular surgery, or both, a 1-year incidence of AF recurrence of 18% was reported in patients with POAF.^9^

The SWEDEHEART registry documents all patients undergoing cardiac surgery in Sweden. When combined with other mandatory national registries, it has the capacity to deliver complete follow-up data, with registration of any hospitalization and hospital-affiliated outpatient contact enabling the calculation of a cumulative incidence of AF recurrence in postsurgical populations. The cumulative incidence of 10.0% at 1 year observed in this study is near the 8.7% rate reported by Butt et al.^23^ After 14 years, the cumulative incidence of AF recurrence in our population was 33.1%. It is noteworthy that the episodes reported in our study are clinically relevant episodes leading to hospitalization or treatment at a hospital outpatient clinic. In a study comparing 30-day continuous electrocardiogram monitoring with standard of care, the underreporting of subclinical POAF in cardiac surgery patients was highlighted by a 17.9% absolute difference in AF detection rate.^24^ Using continuous cardiac rhythm monitoring, 2 studies demonstrated that both the incidence of POAF and the AF recurrence rate after CABG are most likely underestimated.^25,26^

Several studies have documented associations between POAF after CABG and increased risk of complications. Postoperative AF has been associated with an increased risk of all-cause mortality,^2,27^ ischemic stroke,^4^ thromboembolism,^5^ heart failure,^5^ and bleeding^28,29^ after CABG. The question arises regarding whether POAF is causally related to these negative outcomes or whether a further driver (eg, AF recurrence) may be the substitute associated with worse prognosis. We were unable to identify an association between early AF recurrence and all-cause mortality, ischemic stroke, or any thromboembolism. Conversely, we identified an association between early AF recurrence and heart failure hospitalization after discharge.

In an analysis of data from the Framingham Heart Study, researchers reported that “AF occurs in more than half of individuals with heart failure, and heart failure in more than one-third of individuals with AF.”^30^ The association between heart failure and AF is further underpinned by the similarity of the risk factors of the 2 conditions. When these conditions co-occur, they confer substantially increased mortality^31^ compared with independent occurrence. In a previous analysis of patients in the SWEDEHEART registry who underwent CABG, which was based on partly the same study population as our study, POAF was associated with an increased risk of heart failure hospitalizations (AHR, 1.35 [95% CI, 1.21-1.51]).^5^ In this study of patients who underwent CABG, we observed that early AF recurrence after POAF was associated with heart failure hospitalizations (AHR, 1.80 [95% CI, 1.32-2.45]). Note that in our analysis, only heart failure events occurring after recurrence events of AF were included in the calculation of hazard ratios. Analogously, researchers in the Framingham Heart Study found that AF more frequently antedated heart failure events than the reverse.^30^ Our results suggest that patients with clinically relevant early AF recurrence after CABG constitute a risk group who need close surveillance with a focus on preventing and treating heart failure.

Current guidelines propose that it is reasonable to treat patients with POAF with oral anticoagulation in a similar manner as nonsurgical patients.^32^ Furthermore, guidelines recommend that the benefits and risks of anticoagulation therapy should be weighed individually.^33^ However, studies investigating oral anticoagulation in patients with POAF after CABG in the Society of Thoracic Surgeons Adult Cardiac Surgery Database suggest no net clinical benefit^28^ and even a possible increased mortality risk.^29^ In this study, we observed, as expected, that patients were more likely to receive oral anticoagulation therapy after recurrence events. Three months after recurrence, 90.0% of patients with early AF recurrence had been dispensed an oral anticoagulant compared with 31.9% of patients without AF recurrence. Furthermore, we observed an association between AF recurrence and major bleeding events, likely attributable to oral anticoagulation, often in combination with antiplatelet therapy.

### Strengths and Limitations

To our knowledge, this study represents the first investigation of the association between early AF recurrence and outcome events in patients with POAF after CABG. Strengths of this study include the large study population, the long follow-up, the use of validated registries, and the completeness of the data. Like all studies investigating POAF, a key limitation is the definition of POAF. Although the present definition is valid and verifiable within the registry used, a comparison with other studies must be performed carefully because of variability in the definition. We acknowledge that this study only investigated patients with symptomatic AF recurrence, and patients with silent recurrence were not included. An investigation of patients with silent AF necessitates the use of continuous monitoring devices. Use of administrative data may underestimate the incidence of AF. However, it has previously been shown that the sensitivity and specificity for detecting AF in registries based on *ICD* codes is reasonable.^34^ A small portion of the patients investigated may have been in AF at discharge; with the available data, it was not possible to determine whether or when conversion occurred. Patient-level data on combined antiplatelet and oral anticoagulant use, as well as use at the time of the investigated outcome events, was not available within our database. Multiple comparisons increase the risk of false-positive findings; however, this was unlikely in our study, given the robust *P* values for heart failure hospitalization and major bleeding. Finally, as in all observational studies, there remains a risk for selection bias and residual confounding in our study (although the E-value analysis indicated moderate robustness).

## Conclusions

In this cohort study, early AF recurrence after discharge in patients with new-onset POAF after CABG was associated with subsequent heart failure necessitating hospital care, an increased burden of oral anticoagulation, and an increased risk of major bleeding events. These results suggest that close follow-up of patients with POAF and early AF recurrence is required.

## Notes

Supplement 1. **eTable 1.** List of Diagnoses According to *International Classification of Diseases*, *Tenth Revision* (*ICD-10*) Codes **eTable 2.** List of Medications According to ATC Codes **eTable 3.** Variables Included in the Individual Multivariable Cox Proportional Hazards Models Investigating the Association of Early Atrial Fibrillation Recurrence With Outcomes **eTable 4.** Cumulative Incidence of Atrial Fibrillation Recurrence After Discharge in Patients With New-Onset Postoperative Atrial Fibrillation After Coronary Artery Bypass Grafting **eTable 5.** Sensitivity Analysis for the Association of Early Atrial Fibrillation Recurrence With Primary and Secondary Outcome Variables **eMethods.** Sensitivity Analysis **eFigure.** Atrial Fibrillation Recurrence in Patients With New-Onset Postoperative Atrial Fibrillation After Coronary Artery Bypass Grafting With Grouping by Year of Surgery

Supplement 2.
Data Sharing Statement
