# Supplementary material for: Recurrence of Atrial Fibrillation in Patients With New-Onset Postoperative Atrial Fibrillation After Coronary Artery Bypass Grafting
Source: JAMA Netw Open. 2024 Mar 7;7(3):e241537. doi: 10.1001/jamanetworkopen.2024.1537 (PMC10921254; doi:10.1001/jamanetworkopen.2024.1537)
Supplement: Supplement 1. — eTable 1. List of Diagnoses According to International Classification of Diseases, Tenth Revision (ICD-10) Codes eTable 2. List of Medications According to ATC Codes eTable 3. Variables Included in the Individual Multivariable Cox Proportional Hazards Models Investigating the Association of Early Atrial Fibrillation Recurrence With Outcomes eTable 4. Cumulative Incidence of Atrial Fibrillation Recurrence After Discharge in Patients With New-Onset Postoperative Atrial Fibrillation After Coronary Artery Bypass Grafting eTable 5. Sensitivity Analysis for the Association of Early Atrial Fibrillation Recurrence With Primary and Secondary Outcome Variables eMethods. Sensitivity Analysis eFigure. Atrial Fibrillation Recurrence in Patients With New-Onset Postoperative Atrial Fibrillation After Coronary Artery Bypass Grafting With Grouping by Year of Surgery [file jamanetwopen-e241537-s001.pdf]

Supplementary Online Content

Herrmann FEM, Taha A, Nielsen SJ, et al. Recurrence of atrial fibrillation in patients with new-onset postoperative atrial fibrillation after coronary artery bypass grafting. *JAMA Netw Open*. 2024;7(3):e241537. doi:10.1001/jamanetworkopen.2024.1537

eTable 1. List of Diagnoses According to *International Classification of Diseases*, *Tenth Revision* (*ICD-10*) Codes
eTable 2. List of Medications According to ATC Codes
eTable 3. Variables Included in the Individual Multivariable Cox Proportional Hazards Models Investigating the Association of Early Atrial Fibrillation Recurrence With Outcomes
eTable 4. Cumulative Incidence of Atrial Fibrillation Recurrence After Discharge in Patients With New-Onset Postoperative Atrial Fibrillation After Coronary Artery Bypass Grafting
eTable 5. Sensitivity Analysis for the Association of Early Atrial Fibrillation Recurrence With Primary and Secondary Outcome Variables
eMethods. Note on Sensitivity Analysis
eFigure. Atrial Fibrillation Recurrence in Patients With POAF After CABG With Grouping by Year of Surgery

This supplementary material has been provided by the authors to give readers additional information about their work.

© 2024 Herrmann FEM et al. *JAMA Network Open.*

eTable 1. List of Diagnoses According to *ICD-10* Codes

Variable	ICD-codes
Intracranial bleeding	I60, I61, I62, I690, I691, I692
Gastrointestinal bleeding	I850, I983, K226, K250, K252, K254, K256, K260, K262, K264, K266, K270, K272, K274, K276, K280, K284, K290, K625, K661, K920, K921, K922, K25, K26, K27, K28, I850, I983, K221, K226
Hemopericardium	I230, I312
Hemothorax	J942
Urogenital bleeding	N02, R319, N95, N939, N501A
Other bleeding	H431, R04, R58, D629, T810, DR029, D50
Anemia	D50-64
Atrial fibrillation	I48
Ischemic stroke	I63, I69.3, I69.4
Stroke unspecified	I64
TIA	G45, I66, I65
Peripheral arterial embolism	I74
Heart failure	I50, I110, I130, I132, I255, I42-43
Hypertension	I10-15
Diabetes mellitus	E10-14
Ischemic heart disease	I20, I24, I25
Myocardial infarction	I21, I22
PCI	FNG
Peripheral vascular disease	I70, I71, I72, I73, I74, I77
Vascular disease	I21, I22, I252, I70-73
Renal disease and RRT	N17-19, DR016, DR024, KAS10, KAS20
Liver disease	K70-77, JJB, JJC
Alcohol	E244, F10, G312, G621, G721, I426, K292, K70, K860, O354, P043, Q860, T51, Y90-91, Z502, Z714
COPD	J44
Pulmonary embolism	I26
DVT	I80

COPD: Chronic obstructive pulmonary disease; DVT: Deep vein thrombosis; ICD-10: International classification of diseases 10th version; PCI: Percutaneous coronary intervention; RRT: Renal replacement therapy; TIA: Transitory ischemic attack

© 2024 Herrmann FEM et al. *JAMA Network Open.*

eTable 2. List of Medications According to ATC Codes

Medication	ATC-codes
Renin-angiotensin inhibitors	C09
Betablockers	C09 (excluding C07AA07)
Mineralocorticoid receptor antagonists	C03DA
Calcium antagonists	C08
Oral anticoagulants	B01AA, B01AE, B01AF
Antiplatelets	B01AC, N02bA
Lipid lowering agents	C10
Antidiabetics	A10
Systemic corticosteroids	H02
Anti-inflammatory and antirheumatic agents	M01
Diuretics	C03 (excluding C03DA)
Antiarrhythmic drugs	C01B, C07AA07
Digoxin	C01AA05

ATC: The Anatomical Therapeutic Classification

© 2024 Herrmann FEM et al. *JAMA Network Open.*

eTable 3. Variables Included in the Individual Multivariable Cox Proportional Hazards Models Investigating the Association of Early Atrial Fibrillation Recurrence With Outcomes

Outcome investigated	Variables included in model
All-cause mortality	1. Atrial fibrillation recurrence<br>2. Gender<br>3. Age at the time of CABG<br>4. History of diabetes mellitus<br>5. History of hypertension<br>6. History of chronic respiratory disease<br>7. History of congestive heart disease<br>8. History of cancer<br>9. CHA_2DS_2-VASc score over 4 points<br>10. History of ischemic stroke<br>11. History of transitory ischemic attack<br>12. History of liver disease<br>13. History of pulmonary embolism<br>14. History of deep vein thrombosis<br>15. History of systemic embolism
Ischemic Stroke	1. Atrial fibrillation recurrence<br>2. Gender<br>3. Age at the time of CABG<br>4. Estimated glomerular filtration rate at the time of CABG<br>5. History of diabetes mellitus<br>6. History of hypertension<br>7. History of cancer<br>8. History of transitory ischemic attack<br>9. History of pulmonary embolism<br>10. History of systemic embolism
Any thromboembolism	1. Atrial fibrillation recurrence<br>2. Gender<br>3. Age at the time of CABG<br>4. Estimated glomerular filtration rate at the time of CABG<br>5. History of diabetes mellitus<br>6. History of hypertension<br>7. History of cancer<br>8. CHA_2DS_2-VASc score over 4 points<br>9. History of ischemic stroke<br>10. History of transitory ischemic attack<br>11. History of pulmonary embolism<br>12. History of systemic embolism
Heart failure hospitalization	1. Atrial fibrillation recurrence<br>2. Gender<br>3. Age at the time of CABG<br>4. History of cardiomyopathy<br>5. History of congestive heart disease<br>6. History of hypertension<br>7. History of myocardial infarction<br>8. History of cancer

© 2024 Herrmann FEM et al. *JAMA Network Open.*

**Outcome investigated**	**Variables included in model**
Major Bleeding	1. Atrial fibrillation recurrence<br>2. Gender<br>3. Age at the time of CABG<br>4. History of hypertension<br>5. History of hemorrhagic stroke<br>6. History of oral anticoagulation<br>7. History of renal failure<br>8. History of antiplatelet therapy<br>9. History of subdural bleeding<br>10. History of gastrointestinal bleeding<br>11. History of intracranial bleeding<br>12. History of myocardial infarction

CABG: Coronary artery bypass grafting
CHA_2DS_2-VASc: score used to stratify risk of stroke in patients with atrial fibrillation based on the following characteristics – congestive heart failure, hypertension, age, diabetes mellitus, stroke/transitory ischemic attack, vascular disease and sex category

© 2024 Herrmann FEM et al. *JAMA Network Open.*

eTable 4. Cumulative Incidence of Atrial Fibrillation Recurrence After Discharge in Patients With New-Onset Postoperative Atrial Fibrillation After Coronary Artery Bypass Grafting

Time after Discharge	Cumulative Incidence[a] of AF Recurrence (95% CI)
**Months after Discharge**	
0	0
1	3.8 (3.5-4.2)
3	6.7 (6.2-7.1)
6	8.4 (7.9-8.9)
9	9.3 (8.8-9.9)
**Years after Discharge**	
1	10.0 (9.5-10.6)
2	12.1 (11.4-12.7)
3	14.2 (13.5-14.9)
4	15.9 (15.2-16.7)
5	18.1 (17.3-18.8)
6	19.8 (19.0-20.6)
7	21.5 (20.6-22.3)
8	23.3 (22.3-24.2)
9	25.2 (24.2-26.2)
10	26.8 (25.8-27.9)
11	28.4 (27.3-29.6)
12	29.4 (28.2-30.6)
13	31.4 (30.0-32.8)
14	33.1 (31.1-35.2)

[a]Estimated via a competing risk regression to account for the competing risk of death
AF: Atrial fibrillation

© 2024 Herrmann FEM et al. *JAMA Network Open.*

eTable 5. Sensitivity Analysis for the Association of Early Atrial Fibrillation Recurrence With Primary and Secondary Outcome Variables

Outcomes	Unadjusted HR (95% CI) Early AF Recurrence[a] vs. No Recurrence	Adjusted[b] HR (95% CI) Early AF Recurrence vs. No Recurrence	P Value
All-cause mortality	1.09 (0.73-1.62)	1.14 (0.77-1.70)	.51
Ischemic stroke	0.65 (0.37-1.14)	0.73 (0.42-1.28)	.27
Any thromboembolism	0.59 (0.36-0.97)	0.65 (0.39-1.07)	.09
Heart failure hospitalization	1.76 (1.28-2.42)	1.74 (1.24-2.45)	.001
Major bleeding	1.58 (1.13-2.21)	1.68 (1.20-2.36)	.003

AF: atrial fibrillation. HR: hazard ratio (calculated for the first 2 postoperative years). aRecurrence within 3 months of discharge. bAdjusted for patient characteristics and comorbidities (identical adjustments as in primary analysis).

© 2024 Herrmann FEM et al. *JAMA Network Open.*

eMethods. Sensitivity Analysis

The sensitivity analysis was performed basing the Cox proportional hazard computations on data prepared in a format differing from that used in primary analysis. This was done by adding blanking periods in each group. Only outcome events occurring after AF recurrence were considered in the early AF recurrence group. Only outcome events occurring later than 21 days after discharge (median time to early AF recurrence) were considered in patients in the no early AF recurrence group.

eFigure. Atrial Fibrillation Recurrence in Patients With New-Onset Postoperative Atrial Fibrillation After Coronary Artery Bypass Grafting With Grouping by Year of Surgery

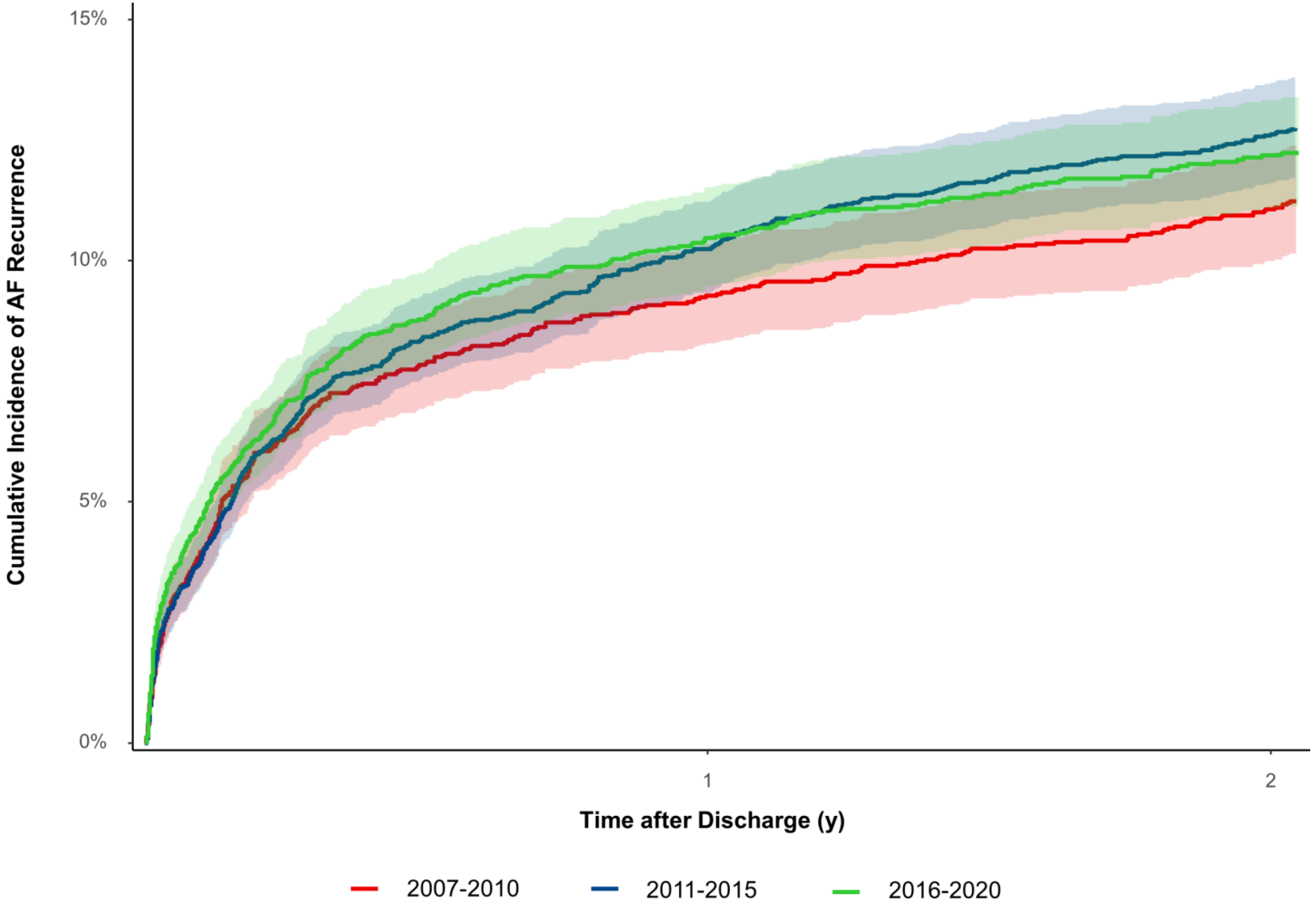

Competing risks regression indicating the cumulative incidence of atrial fibrillation recurrence (with a competing risk of death) in patients with new-onset postoperative atrial fibrillation after coronary artery bypass grafting. Patients are grouped by the year when surgery was performed. The error bands represent 95 % confidence intervals.

© 2024 Herrmann FEM et al. *JAMA Network Open.*
