# Supplementary material for: Recurrence of Atrial Fibrillation in Patients With New-Onset Postoperative Atrial Fibrillation After Coronary Artery Bypass Grafting
Source: JAMA Netw Open. 2024 Mar 7;7(3):e241537. doi: 10.1001/jamanetworkopen.2024.1537 (PMC10921254; doi:10.1001/jamanetworkopen.2024.1537)
Supplement: Supplement 2. — Data Sharing Statement [file jamanetwopen-e241537-s002.pdf]

Data Sharing Statement

Herrmann. Recurrence of Atrial Fibrillation in Patients With New-Onset Postoperative Atrial Fibrillation After Coronary Artery Bypass Grafting. *JAMA Netw Open*. Published March 07, 2024. doi:10.1001/jamanetworkopen.2024.1537

Data
Data available: Yes
Data types: Deidentified participant data
How to access data: Contact the corresponding author (anders.jeppsson@vgregion.se)
When available: With publication

Supporting Documents
Document types: None

Additional Information
Who can access the data: Anyone
Types of analyses: Research purpose.
Mechanisms of data availability: Investigator support after approval of a proposal.
Any additional restrictions: Permission from Swedish Research Review Authority, SWEDEHEART and the The National Board of Health and Welfare is requested.
